# The mechanisms and strategies for Alzheimer’s disease prevention: An update

**DOI:** 10.1016/j.apsb.2026.04.007

**Published:** 2026-04-17

**Authors:** Mingfeng Han, Nuoya Wang, Mengxiu Song, Chenfei Liu, Yedan Wu, Jishan Yin, Lili Jin, Wenyu Jin, Zhonggao Gao

**Affiliations:** a School of Pharmacy, Yanbian University, Yanji 133000, China; b State Key Laboratory of Bioactive Substance and Function of Natural Medicines, Department of Pharmaceutics, Institute of Materia Medica, Chinese Academy of Medical Sciences and Peking Union Medical College, Beijing 100050, China; c Department of Respiratory and Critical Care Medicine, Tianjin Chest Hospital, Tianjin 300000, China; d Department of Dermatology, Yanbian University Hospital, Yanji 133000, China

**Keywords:** Alzheimer’s disease, Geriatric medicine, Prevention strategies, Biomarkers, Public health policy, Lifestyle intervention, Trace elements, Vitamin

## Abstract

Alzheimer’s disease (AD), a progressive neurodegenerative disorder, poses growing global health and socioeconomic challenges due to aging populations and limited therapeutic efficacy. Current treatments, including cholinesterase inhibitors and anti-amyloid monoclonal antibodies, can only delay disease progression without reversing pathology. Emphasizing on prevention, this review provides key updates on advancements in the pathogenesis, diagnosis, and intervention of AD highlighting preventive strategies that can target modifiable risk factors. Key findings underscore the role of managing hypertension and diabetes, optimizing trace elements, vitamins, and regular physical exercise in mitigating the risk of AD. Biomarker-based early diagnosis and emerging therapies provide further support for proactive intervention. Future challenges include the long-term validation of preventive measures and policy-driven funding for large-scale cohort studies. Prioritizing prevention through lifestyle modifications, nutritional balance, and precision medicine is pivotal to reduce the burden of AD in aging societies.

## Graphical abstract

This review highlights prevention strategies for Alzheimer’s disease, including managing hypertension and diabetes, optimizing nutrition and exercise, and leveraging biomarker-based early diagnosis. Prioritizing lifestyle and precision medicine is key to reducing AD burden.

**Figure:**
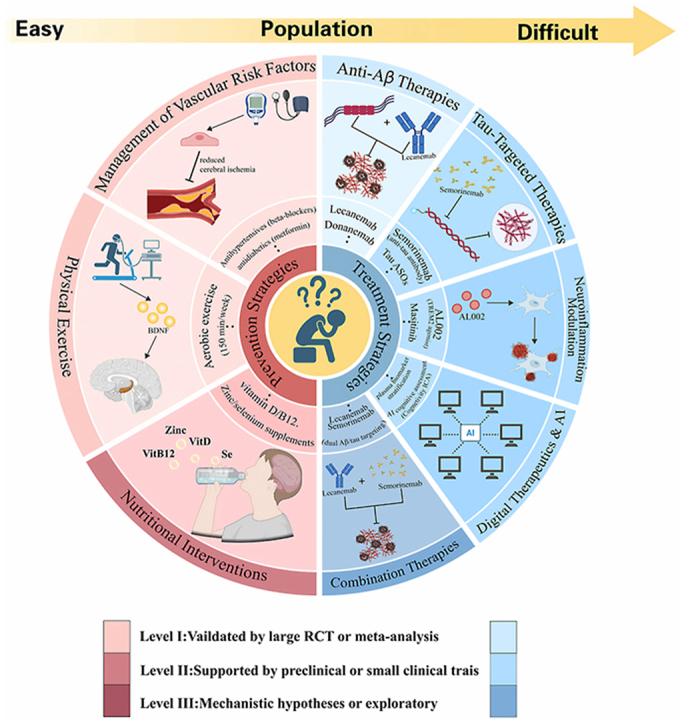

## 1 Introduction

Alzheimer’s disease (AD) represents a profound challenge in neurodegenerative medicine, characterized by progressive cognitive decline and complex pathophysiology. As global demographics shift toward older populations, AD prevalence continues to rise, driving urgent research efforts across medical and neuroscientific disciplines^1^^,^^2^. Central to AD pathology are two hallmark processes: the extracellular accumulation of beta-amyloid into senile plaques and the intracellular formation of neurofibrillary tangles from hyperphosphorylated tau protein^3^^,^^4^. These pathological changes ultimately drive synaptic dysfunction and neuronal loss, underpinning the disease’s clinical progression^5^.

From an epidemiological perspective, the high incidence of AD inflicts considerable distress upon individual patients and imposes a substantial burden on families and society^6^. At the familial level, providing care for patients with AD demands a considerable investment of human, material and temporal resources, and family members frequently encounter long-term psychological pressure and emotional exhaustion. At the societal level, as the number of patients keeps rising continuously, the demand for medical resources, long-term care facilities and an appropriate social security system has escalated sharply, thus presenting severe challenges to the public health system and the social economy^7^.

The current treatment strategies for AD mainly include pharmacological treatment and non-pharmacological intervention. In terms of pharmacological treatment, cholinesterase inhibitors and *N*-methyl-d-aspartate (NMDA) receptor antagonists represent the two major types of therapeutic drugs. Cholinesterase inhibitors increase the concentration of acetylcholine in the brain by inhibiting the activity of acetylcholinesterase to partially compensate for the deficiency of cholinergic neurotransmitter transmission caused by neuronal damage; these inhibitors can exert a certain improvement effect on cognitive function during the early stages of AD^8^^,^^9^. NMDA receptor antagonists can relieve some symptoms in patients with moderate to severe AD by regulating glutamatergic neurotransmission and reducing excitotoxic damage to neurons^10^. However, these pharmacological treatment regimens can only delay the progression of disease for a limited period and cannot fundamentally prevent or reverse the pathological process of AD. Non-pharmacological intervention measures, such as cognitive training, rehabilitation care, music therapy and social activities, can improve the cognitive function and quality-of-life of patients to a certain extent. Due to the lack of standardized intervention protocols and systems to evaluate long-term efficacy, the sustainability and stability of non-pharmacological interventions remain controversial. Currently, all treatment methods aim to slow the progression of AD; however, cannot reverse the disease^11^.

The limited efficacy of current AD treatments, coupled with the disease’s substantial personal and societal burden, has spurred intense interest in prevention. Interventions initiated preclinically or at the earliest disease stages, such as lifestyle modification, risk factor management, and cognitive support, could potentially alter AD trajectory, lowering incidence, delaying onset, and mitigating its multifaceted impact. Embracing prevention aligns with contemporary medicine’s shift toward proactive health and is fundamental to addressing AD as a global health priority. This paradigm also opens new avenues for developing integrated prevention-therapy frameworks.

This review synthesizes key advances from the last five years across AD pathogenesis, diagnostics, therapeutics, and centrally prevention. We focus specifically on translating prevention evidence into actionable strategies for at-risk populations. By consolidating this evidence, our goals are threefold: to inform public health policy, to guide future research priorities, and to support clinical practices aimed at preemptive management. As the AD landscape evolves, refining and implementing evidence-based prevention will be essential to alleviating disease burden and preserving patient quality of life.

## 2 The pathogenic mechanism of AD

AD arises from a multifactorial pathogenesis that remains incompletely understood. Currently, the core mechanisms that have been extensively studied and recognized predominantly revolve around the beta-amyloid cascade hypothesis and the tau protein hypothesis^12^^,^^13^.

The Abeta cascade hypothesis posits that the abnormal metabolism and aggregation of Abeta in the brain is the key initiating event in the pathogenesis of AD. Under physiological conditions, the production and clearance of Abeta are in equilibrium^14^. However, due to genetic factors (such as certain gene mutations that can result in the abnormal cleavage of the Abeta precursor protein [APP]), environmental factors (such as head trauma and heavy metal exposure that can affect the metabolic process of Abeta), and age-related physiological changes (such as the decline in proteasome function and abnormal autophagy in the brain, leading to impaired Abeta clearance), excessive Abeta is produced or its clearance is reduced, gradually accumulating in the brain to form senile plaques. The accumulation of Abeta is a neurotoxic process and can trigger a series of pathological reactions, including the activation of microglia and astrocytes, leading to neuroinflammatory responses, and the release of a large number of inflammatory factors such as tumor necrosis factor-alpha (TNF-*α*), interleukin-1beta (IL-1*β*), and interleukin-6 (IL-6), causing further damage to neuronal and synaptic functions^15^. Simultaneously, Abeta can also induce oxidative stress responses, generating a large amount of reactive oxygen species (ROS) and reactive nitrogen species, damaging the integrity of cell membranes, impairing mitochondrial functionality, and affecting intracellular signaling pathways, ultimately leading to neuronal apoptosis and death^16^^,^^17^.

The tau protein hypothesis focuses on the role of abnormal changes in tau protein in the pathogenesis of AD^18^^,^^19^. Normally, this microtubule-associated protein stabilizes neuronal microtubules, which is essential for maintaining cell structure and facilitating processes like axonal transport. In AD, however, tau becomes hyperphosphorylated, a shift prompted by factors including Abeta toxicity, genetic mutations, and altered phosphatase activity^20^. This pathological modification causes tau to detach from microtubules and form intracellular neurofibrillary tangles (NFTs). Importantly, not all tau isoforms contribute equally; recent work using human iPSC-derived neurons showed that the 1N4R isoform markedly increases neuronal vulnerability to amyloid-beta and phosphorylated tau^21^. This finding implies that specific tau isoforms can critically compromise synaptic resilience and accelerate disease, mechanistically explaining why certain neuronal populations are selectively targeted in AD. Once formed, NFTs disrupt intracellular trafficking and signaling, impairing neuronal function and ultimately promoting cell death. The accumulation and spread of NFTs throughout the brain are strongly correlated with both clinical symptom progression and the extent of cognitive decline.

Beyond the classical Abeta and tau pathways, emerging research underscores the critical contribution of neurovascular unit (NVU) dysfunction to AD pathogenesis. The NVU, an interactive ensemble of neurons, glia (astrocytes and microglia), and cerebrovascular endothelial cells, orchestrates key brain homeostatic functions, including cerebral blood flow regulation, blood-brain barrier (BBB) integrity, and nutrient-waste exchange^22^. In AD, this coordinated system breaks down, characterized by endothelial damage, BBB hyperpermeability, cerebral hypoperfusion, and perivascular glial activation. Notably, glycocalyx dysregulation on endothelial surfaces has been identified as a mechanism that aggravates BBB impairment with aging and neurodegeneration, promoting neurotoxin influx and Abeta deposition^23^. Collectively, these NVU deficits disrupt the cerebral microenvironment, impairing neuronal metabolism and function while hampering the clearance of Abeta and pathological tau. A compromised BBB, for instance, permits infiltrating peripheral inflammatory mediators to exacerbate neuroinflammation. Concurrent cerebral hypoperfusion induces hypoxia and energy deficits, increasing neuronal vulnerability. Activated perivascular glia further compound damage by releasing inflammatory and neurotoxic factors^24^^,^^25^. Accumulating evidence now positions neurovascular dysfunction not merely as a concurrent phenomenon but as an active contributor to both Abeta and tau pathology^26^, ^27^, ^28^, ^29^. Specifically, BBB disruption allows peripheral inflammatory mediators and proteins like fibrinogen to enter the brain, where they can directly promote Abeta aggregation and stimulate microglial activation. Concurrently, chronic cerebral hypoperfusion hampers the clearance of interstitial Abeta by compromising perivascular drainage, leading to accelerated amyloid plaque deposition. Furthermore, vascular injury itself can induce oxidative stress and excitotoxicity; these secondary insults then activate kinases including GSK-3beta and CDK5, which drive tau hyperphosphorylation^30^. Taken together, this evidence underscores that vascular pathology acts synergistically with traditional mechanisms to exacerbate AD lesions, revealing vascular health as a critical modifiable target for AD prevention.

Genetic predisposition constitutes another critical layer in the pathogenesis of AD. Clear monogenic forms of early-onset familial AD are linked to mutations in genes such as *APP*, *PSEN1* (presenilin 1), and *PSEN2* (presenilin 2)^31^, ^32^, ^33^, ^34^, ^35^. These mutations directly alter Abeta production or processing, leading to its pathological aggregation and disease initiation. In contrast, the genetics of late-onset AD are polygenic and complex. Here, the ε4 allele of the apolipoprotein E (*APOE*) gene stands out as the most significant genetic risk factor. The *APOE ε*4 allele is implicated in multiple pathological pathways, including dysregulated Abeta metabolism, tau hyperphosphorylation, and impaired neurovascular unit function, which collectively elevate an individual’s susceptibility to AD^36^.

AD pathology unfolds over a prolonged preclinical period, often spanning decades before clinical symptoms emerge. Longitudinal studies reveal a characteristic sequence: Abeta deposition commences 15–20 years pre-symptom, stabilizing at a plateau during the preclinical phase. Subsequently, tau hyperphosphorylation and aggregation become detectable around 10–15 years before dementia diagnosis, a timeline that correlates strongly with ensuing neuronal loss and cognitive decline^37^. This evolving pathology is reflected in sequential biomarker changes. Cerebrospinal fluid (CSF) levels of Abeta42 decline nearly twenty years before clinical onset, while phosphorylated tau (p-tau181) and total tau concentrations begin to increase approximately 10–12 years pre-diagnosis. Indicators of neurodegeneration, like hippocampal atrophy observed on MRI or rising plasma neurofilament light chain (NfL), typically manifest nearer to the prodromal and mild cognitive impairment (MCI) stages, signaling more advanced, often irreversible neuronal injury^38^^,^^39^.

This established temporal framework underscores the stage-dependent nature of both pathology and biomarkers. It advances the notion that biomarker profiles can guide stratified prevention and early intervention. For instance, detecting amyloid *via* positron emission tomography (PET) can identify at-risk individuals in the preclinical stage, whereas rising levels of p-tau and NfL may signal impending transition to symptomatic disease, thereby enabling more precisely timed preventive measures^40^, ^41^, ^42^.

## 3 Advances in the diagnosis of AD

Recent years have witnessed a transformation in the diagnostic landscape for AD, propelled by convergent advances in biomarker science, neuroimaging, and artificial intelligence. This progress has shifted focus toward identifying AD in its earliest phases, the preclinical and prodromal stages, where accurate detection is now considered paramount for initiating timely and potentially disease-modifying interventions.

### 3.1 Biomarker-based diagnostic frameworks

The 2024 National Institute on Aging-Alzheimer’s Association (NIA-AA) guidelines emphasize that core biomarker abnormalities (Abeta, tau and neurodegeneration) are sufficient for the diagnosis of AD, enabling earlier and more precise identification of pathology^43^. Cerebrospinal fluid (CSF) biomarkers, including Abeta42/40 ratio, phosphorylated tau (p-tau181) and total tau (t-tau), remain as gold-standard tools (Table 1).

**Table 1:** Early diagnostic modalities for AD: primary use, advantages and limitations.

Modality	Primary use	Advantage	Limitation
Plasma p-tau217 (±Abeta42/40, NfL)	Screening & enrichment; rule-in amyloid/tau	Scalable; cost-effective; correlates with PET	Assay standardization; comorbidity effects
CSF Abeta42/40 and p-tau	Diagnostic confirmation; staging	High accuracy; precedes symptoms	Invasive; access varies
Amyloid PET	Confirm amyloid in atypical/uncertain cases	Visualizes amyloid	Cost; availability; AUC-guided indications
Tau PET	Stage and phenotype; differential diagnosis	Regional mapping	Tracer access; evolving use
Structural/functional MRI	Rule out other causes; atrophy/vascular injury	Non-invasive; widely available	Specificity limited

Abeta, amyloid beta; BBM, blood-based biomarker; CSF, cerebrospinal fluid; PET, positron emission tomography; MRI, magnetic resonance imaging; NIA-AA, National Institute on Aging-Alzheimer’s Association; AUC, appropriate use criteria. Data summarized from multicenter validation studies, longitudinal biomarker cohorts, revised NIA–AA 2024 criteria, and SNMMI/AA AUC 2025 guidelines.

#### 3.1.1 Blood-based biomarkers (BBMs)

Recent multicenter validations indicate that plasma p-tau217 provides the strongest single-analyte performance among all BBMs for identifying brain amyloid pathology during preclinical/MCI stages, with an AUC of 0.94–0.97 across research and real-world cohorts; performance improves further when combined with Abeta42 (for example, p-tau217/Abeta1-42)^44^. Fully automated (Lumipulse) and high-sensitivity Simoa (ALZpath) platforms show high analytical precision and cross-platform agreement, supporting near-term clinical translation. These platforms are minimally invasive and scalable but are limited by ongoing standardization/harmonization across assays and potential confounding by comorbidities^45^.

#### 3.1.2 CSF biomarkers

Consistent with the 2024 NIA-AA revised criteria, core CSF markers (Abeta42/40, p-tau, t-tau) remain highly informative for ultra-early detection. A large 20-year longitudinal study showed that CSF Abeta and p-tau181 deviate from normal approximately 18 and 11 years before clinical diagnosis, respectively, mapping a practical window for pre-symptomatic intervention. These CSF markers are advantageous due to excellent pathophysiological specificity and staging utility but are limited by invasiveness, pre-analytical handling requirements, and limited suitability for population-level screening^46^.

#### 3.1.3 Plasma Abeta42/40

Automated chemiluminescence and LC–MS/MS approaches have demonstrated robust discrimination of amyloid PET status in memory-clinic cohorts. Furthermore, mass-spectrometry methods can function as an “arbiter” when immunoassays disagree. These assays are objective and increasingly automated but are limited by the fact that pre-analytical variables and assay-specific cutoffs require harmonization^47^^,^^48^.

#### 3.1.4 GFAP and NfL

GFAP appears to rise earliest (astrocytic activation) and can predict incident dementia in population cohorts, while NfL tracks axonal injury but is less disease-specific. Multianalyte panels that combine p-tau, Abeta42/40, GFAP, and NfL can enhance risk stratification in preclinical disease. These panels are sensitive to early change but are limited by susceptibility to non-AD comorbidities and the need for context-specific thresholds^49^^,^^50^.

### 3.2 Imaging modalities and neurovascular insights

Advanced imaging techniques have revolutionized AD diagnostics. Abeta-PET and Tau-PET can directly visualize amyloid plaques and neurofibrillary tangles, achieving sensitivities >90%^51^. Hippocampal atrophy detected by MRI remains a cornerstone for tracking disease progression from MCI to AD. Novel approaches, such as 7T ultra-high-field MRI combined with quantitative susceptibility mapping, can reveal spatial correlations between Abeta aggregation, iron deposition and neurodegeneration, thus providing insights into region-specific vulnerability^52^. Furthermore, neurovascular unit dysfunction, evidenced by blood–brain barrier permeability and cerebral hypoperfusion, has emerged as a diagnostic marker linked to the impairment of Abeta clearance and neuroinflammation^22^^,^^25^. The 2025 updated appropriate use criteria recommend amyloid and tau PET primarily for diagnostically uncertain cases, atypical/early-onset presentations, or to establish eligibility/monitoring for disease-modifying therapies. PET is not recommended for screening asymptomatic individuals. The advantages of imaging modalities include *in vivo* pathology confirmation and staging, although these methods can be limited by cost, access, and radiation exposure, and the fact that integration with BBMs can reduce unnecessary scans^53^.

### 3.3 Innovations driven by artificial intelligence

AI technologies are reshaping AD diagnostics through rapid, scalable, and cost-effective solutions. Deep learning algorithms can analyze structural MRI to quantify hippocampal atrophy with levels of precision that are comparable to expert radiologists. AI platforms such as Integrated Cognitive Assessment (ICA) by Cognetivity can enable real-time cognitive monitoring using 5-min computerized tasks, achieving 85% accuracy in distinguishing early AD from normal aging^54^. Multimodal AI models integrating PET, MRI and plasma biomarkers (*e*.*g*., p-tau181 and GFAP) demonstrate superior diagnostic performance (AUC: 0.94) compared to single-modality approaches^55^. AI can also facilitate predictive modeling; for example, machine learning algorithms trained on longitudinal data from the Australian Imaging, Biomarkers and Lifestyle (AIBL) Study cohort were able to forecast AD progression 6–8 years pre-diagnosis using plasma Abeta42/40 and hippocampal volume trajectories^39^. There are still important challenges to address, such as ensuring AI tools are standardized across diverse populations and effectively validated in real-world clinical environments.

### 3.4 Future directions and challenges

Despite considerable progress, practical limitations continue to constrain the broader use of advanced diagnostics. The expense and limited availability of PET and CSF analysis restrict their application, especially in resource-limited environments. Blood-based biomarkers and portable AI platforms offer a more accessible alternative, yet they demand thorough validation across varied ethnic groups. Moving forward, key priorities include establishing unified diagnostic standards, improving the interpretability of AI models, and incorporating multi-omics data (such as epigenomic and proteomic profiles) to refine personalized risk assessment. The advent of wearable technologies for ongoing cognitive and biomarker tracking may also pave the way for more dynamic disease management.

Ultimately, the integration of biomarkers, advanced neuroimaging, and AI is redefining AD diagnostics, shifting the paradigm from post-symptom identification to pre-symptom risk detection. This enhanced capability supports more accurate diagnosis and creates critical opportunities for early clinical intervention, a principle that lies at the heart of the preventive strategies discussed throughout this review.

## 4 Advances in the treatment of AD

**Figure 1:**
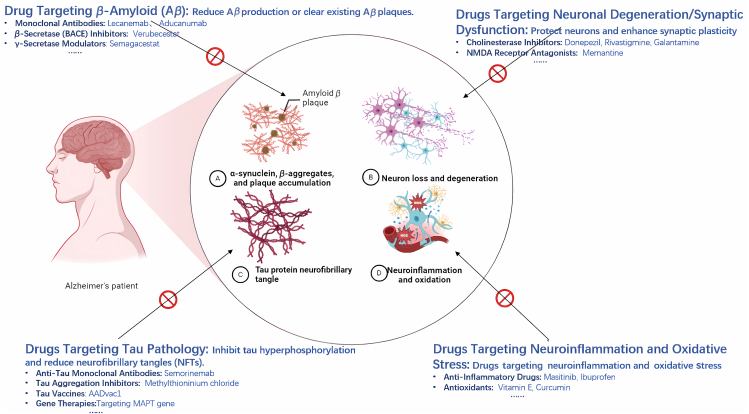
The current main therapeutic mechanisms for Alzheimer’s disease.

The field of AD therapy has advanced considerably in recent years, as new treatments increasingly address fundamental disease processes like Abeta buildup, tau abnormalities, neuroinflammation, and metabolic imbalances^8^^,^^56^. Standard drug options, such as cholinesterase inhibitors (*e*.*g*., donepezil) and NMDA receptor blockers (*e*.*g*., memantine), still form the backbone of symptom control, but their inability to arrest disease advancement has spurred extensive efforts toward disease-modifying therapies (DMTs)^57^. The following sections outline major breakthroughs in AD management, grounded in up-to-date clinical and preclinical evidence (Fig. 1).

### 4.1 Anti-amyloid monoclonal antibodies

The US Food and Drug Administration 2023 approval of lecanemab (Leqembi) represented a landmark event in AD drug development^58^^,^^59^. This humanized antibody targets soluble Abeta protofibrils and reduces amyloid plaque load. The Phase III Clarity AD trial demonstrated that lecanemab slowed cognitive decline by 27% over 18 months when compared to a placebo, as measured by the Clinical Dementia Rating-Sum of Boxes (CDR-SB) score^60^^,^^61^. However, concerns regarding amyloid-related imaging abnormalities (ARIA), particularly microhemorrhages and edema, highlight the need for the careful selection and monitoring of patients^62^, ^63^, ^64^. Gantenerumab, an anti-Abeta monoclonal antibody, completed its Phase III GRADUATE trials in 2023; however, failed to meet the primary endpoint (CDR-SB score improvement). Subgroup analyses revealed an 18% slowing of cognitive decline in APOE4 non-carriers, although this drug has not yet received regulatory approval for the treatment of AD^65^^,^^66^. Another anti-Abeta antibody, donanemab, achieved promising results in the TRAILBLAZER-ALZ 2 trial (2023), achieving a 35% reduction in cognitive decline in early patients with AD with high tau pathology^67^. Notably, donanemab targets pyroglutamate-modified Abeta, a highly aggregated form, thus enabling the faster clearance of plaques^68^.

### 4.2 Tau-targeted therapies

Tau-focused therapies aim to inhibit hyperphosphorylation, aggregation or promote the clearance of pathological tau^69^. Treatment with semorinemab, an anti-tau monoclonal antibody, yielded mixed results in Phase II trials, improving cognitive outcomes in mild AD; however, failing in moderate cases^70^. Conversely, MK-2214, an inhibitor of tau aggregation, reduced tau pathology in preclinical models and is advancing to Phase I trials^71^. Gene-silencing approaches, such as antisense oligonucleotides (ASOs) targeting *MAPT* (the tau-encoding gene), have achieved efficacy in reducing the expression of tau in animal models. A Phase I/II trial of IONIS-MAPT Rx is underway to evaluate safety and biomarker changes in patients with AD^72^.

### 4.3 Neuroinflammation modulation

Microglial activation and chronic neuroinflammation are increasingly being recognized as drivers of AD progression^73^. AL002, a monoclonal antibody targeting TREM2, a receptor that regulates microglial function, has been shown to enhance amyloid clearance and improve cognition in preclinical studies. Phase II trials are ongoing^74^. Furthermore, treatmenr with masitinib, a tyrosine kinase inhibitor that modulates mast cells and microglia, achieved a 40% reduction in cognitive decline in a Phase III trial (AB09004) for mild-to-moderate AD^75^. Recent clinical evidence provides critical insights into microglial mechanisms in immunized patients with AD. Research revealed that anti-Abeta immunotherapy (*e*.*g*., lecanemab) enhanced microglial phagocytic activity and the lysosomal degradation of Abeta, particularly in patients with APOE4/4 genotype. This mechanism reduced amyloid burden and attenuated neuroinflammation, thus suggesting that targeting microglial function could synergize with existing immunotherapies to improve clinical outcomes^76^. These findings underscore the potential of combining immunomodulatory agents with anti-Abeta therapies to address amyloid pathology and neuroinflammation in AD.

### 4.4 Gene and metabolic therapies

Gene-editing technologies such as CRISPR-Cas9 are being investigated in an attempt to correct mutations in *APP*, *PSEN1* and *PSEN2* in familial models of AD^77^, ^78^, ^79^. While remaining in preclinical stages, these approaches hold potential for personalized interventions. Metabolic interventions targeting insulin resistance and mitochondrial dysfunction are gaining traction. Intranasal insulin was shown to improve memory in APOE4 carriers in the SNIFF trial, whereas elamipretide, a mitochondrial-targeted peptide, enhanced neuronal energy metabolism in patients with early AD^80^.

### 4.5 Multi-target and combination therapies

Given the multifactorial nature of AD, combination therapies are now under investigation. The DIAN-TU trial (2023) evaluated simultaneous Abeta (gantenerumab) and tau (semorinemab) targeting in autosomal dominant AD, and recorded synergistic effects on biomarker reduction^81^. Similarly, the combination of lecanemab and anti-tau agents is set to be investigated in upcoming trials.

### 4.6 Digital therapeutics and precision medicine

The rapid advancement of AI technology has garnered increasing attention for the diagnosis and therapeutic management of AD. A number of nascent AI technologies have exhibited potential in the rapid diagnosis and treatment of AD. AI-driven platforms, such as the ICA by Cognetivity, enable the real-time monitoring of cognitive changes, thus facilitating early intervention^54^^,^^55^. Blood-based biomarkers (*e*.*g*., plasma p-tau181 and GFAP) are now being used to stratify patients for targeted therapies, exemplified by the AHEAD 3-45 trial, which leverages amyloid PET and plasma biomarkers to identify preclinical AD participants^39^.

### 4.7 Challenges and future directions

Translating these therapeutic advances into widespread clinical practice faces several barriers. The high cost of newer treatments, risks such as amyloid-related imaging abnormalities (ARIA), and unequal access to advanced diagnostic tools remain significant hurdles (Table 2^60^^,^^61^^,^^82^, ^83^, ^84^, ^85^, ^86^, ^87^, ^88^, ^89^, ^90^, ^91^, ^92^, ^93^, ^94^). To solidify their role in care, rigorous large-scale trials evaluating long-term outcomes and effectiveness in diverse, real-world settings are essential. The AD therapeutic pipeline is now burgeoning, marked prominently by the advent of anti-amyloid antibodies. Looking ahead, the most impactful management strategy will probably integrate disease-modifying therapies with principles of precision medicine and evidence-based lifestyle interventions.

**Table 2:** Summary of approved medications for Alzheimer’s disease and associated side effects (as of March 2025).

Drug class	Drug name	Formulation	Indication	Main side effects/issues
Cholinesterase inhibitors	Donepezil	Oral tablet/Transdermal patch	Mild to severe AD	Nausea (30%), vomiting (20%), diarrhea (15%), headache, insomnia, weight loss^82^^,^^83^
	Rivastigmine	Oral capsule/Transdermal patch	Mild to moderate AD	GI disturbances (nausea, vomiting), hepatotoxicity (requires liver monitoring)^84^^,^^85^
	Galantamine	Oral tablet/Extended-release capsule	Mild to moderate AD	Allergic reactions (contraindicated), dizziness, decreased appetite^86^^,^^87^
NMDA antagonists	Memantine	Oral tablet/Solution	Moderate to severe AD	Dizziness (10%), confusion (dose reduction needed), constipation; requires dose adjustment in renal impairment^88^^,^^89^
	Memantine/Donepezil combination	Extended-release capsule	Moderate to severe AD	Combined side effects (both cholinesterase inhibitor and NMDA antagonist risks)^90^
Anti-Abeta monoclonal antibodies	Lecanemab	Intravenous infusion	Early AD	ARIA (amyloid-related imaging abnormalities—edema/hemorrhage, higher risk in APOE4 carriers), infusion reactions^60^^,^^61^
	Donanemab	Intravenous infusion	Early AD	ARIA, headache, increased fall risk; requires regular MRI monitoring^91^^,^^92^
Others	Sodium oligomannate (GV-971)	Oral capsule	Mild to moderate AD	GI discomfort (bloating, diarrhea); limited long-term safety data^93^^,^^94^
	Benzgalantamine (ZUNVEYL)	Extended-release tablet	Mild to moderate AD	Milder GI effects (new extended-release design), but higher cost

In addition to the therapies discussed in this section, several promising candidates in Phase II and III clinical trials are poised to further transform the therapeutic landscape of AD. These therapies target diverse mechanisms, including Abeta and tau pathology, neuroinflammation, synaptic plasticity and metabolic dysregulation. Table 3^95^, ^96^, ^97^, ^98^, ^99^, ^100^, ^101^, ^102^, ^103^, ^104^, ^105^ highlights some of the most advanced and innovative approaches currently under investigation.

**Table 3:** Major ongoing clinical trials for Alzheimer’s disease. This table summarizes major ongoing clinical trials for Alzheimer’s disease, updated with the most recent information available as of August 2025. Data were obtained from official trial registries (ClinicalTrials.gov), interim conference reports (*e*.*g*., AAIC 2025, AD/PD 2025), and published literature. Key findings include preliminary efficacy signals (*e*.*g*., CSF biomarker reduction, cognitive stabilization) and safety profiles.

Therapy	Mechanism	Phase	Key findings/Updates	Estimated completion date	NCT No.	Developer
E2814^95^^,^^96^	Anti-tau (MTBR) monoclonal antibody	Phase II/III	Interim analysis (AAIC 2025): ∼35%–40% reduction in CSF tau aggregates; stable hippocampal volume *vs* placebo	2026 Q2	NCT05399888	Eisai
ALZ-801 (Valiltramiprosate)^97^, ^98^, ^99^	Abeta oligomer inhibitor	Phase III	Updated 2025: APOE4/4 subgroup—cognitive decline slowed by 32% over 18 months; safety profile favorable	2026 Q1	NCT04770220	Alzheon
XPro1595^100^^,^^101^	Soluble TNF inhibitor	Phase II	2025 interim readout: Improved CDR-SB scores, reduced neuroinflammation markers; well tolerated	2025 Q3	NCT06115713	INmune Bio
Anavex 2-73 (Blarcamesine)^102^	Sigma-1 receptor agonist	Phase II/III	Reported at AD/PD 2025: 30% slowing of cognitive decline, improved synaptic function	2025 Q4	NCT03790709	Anavex life sciences
T3D-959^103^	PPAR*δ*/*γ* agonist	Phase II	2025 interim results: Improved brain glucose metabolism, cognitive stabilization in MCI subjects	2025 Q2	NCT05531526	T3D therapeutics
BIIB080 (IONIS-MAPT Rx)^104^	Tau mRNA antisense oligonucleotide	Phase II	2025 interim update: ∼50% reduction in CSF tau; significant executive function improvement	2025 Q3	NCT05399888	Biogen/Ionis
CT1812^105^	Sigma-2 receptor modulator	Phase II	2025 Q1 report: Synaptic density preservation, improved memory/attention; favorable safety	2025 Q1	NCT05531656	Cognition therapeutics

It is notable that the existing treatment regimens for AD mainly aim to retard the progression of disease. Regardless of the intervention at any stage of pathogenesis, it is currently impossible to fundamentally cure AD. Therefore, there is still a need for the continuous exploration and development of more effective treatment strategies and medications.

## 5 Advances in the prevention of AD

Currently, there are no satisfactory therapeutic approaches for AD, and particularly, there is no means to reverse this disease^106^, ^107^, ^108^. Therefore, exploring preventive measures for AD is of paramount importance. In contrast to relatively complex examinations and treatments with more significant side effects, it is more achievable for the common high-risk population of AD to focus on prevention in their daily lives.

### 5.1 Prevention of geriatric diseases

**Figure 2:**
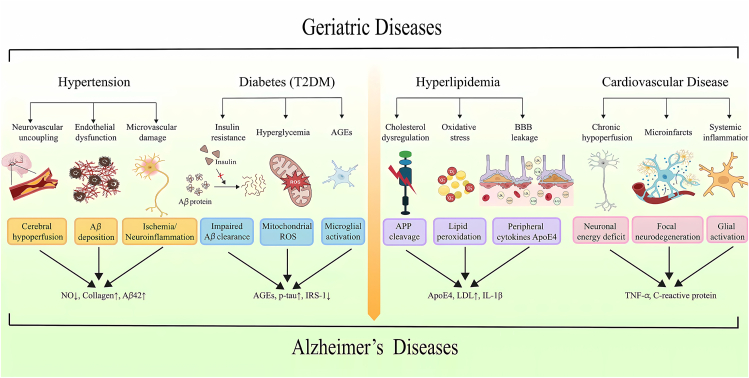
The pathogenic role of geriatric diseases in Alzheimer’s disease.

Many prevalent age-related diseases, such as hypertension, hyperlipidemia, and diabetes mellitus, are prone to lead to secondary senile dementia (Fig. 2)^102^^,^^103^. Preventing the onset and progression of geriatric diseases constitutes a critical component in the management of AD.

Hypertension is a high-risk factor for AD. A longitudinal cohort study indicated that the presence of hypertension and other vascular risk factors is correlated with the deterioration of performance in executive function and attention tests. The reduction of vascular reserve capacity might result in impaired neurovascular coupling, thereby influencing cognitive ability. Furthermore, endothelial dysfunction and microvascular diseases during middle age could play a significant role in the manifestation and severity of cognitive decline in subsequent years^109^. In hypertensive patients, the use of anti-hypertensive beta-blockers was associated with a 57% reduction in the probability of developing dementia, according to a retrospective analysis^110^. Another study summarized that there exists a potential interdependence between collagen deposition and Abeta deposition in cerebral venules. Furthermore, the impaired function of cerebral venules could result in a reduction of cerebral perfusion pressure; this may give rise to cerebral ischemia, edema and neuroinflammatory responses, and subsequently lead to the occurrence or exacerbation of AD^111^.

A previous retrospective analysis revealed that the administration of metformin in patients with type 2 diabetes (T2D) could reduce the incidence of dementia^112^. In addition, a Mendelian randomization study encompassing 527,138 individuals revealed that the utilization of metformin might reduce the risk of AD in the general population^113^. Another retrospective analysis, encompassing 1,815,032 patients, also discovered that the utilization of anti-hyperglycemic medications (A-HgM) in patients with T2D could reduce the incidence of AD^114^. These research outcomes indicate that diabetes and dementia might share certain pathogenic mechanisms.

Unlike the paucity of therapeutic approaches for AD, therapeutic measures for hypertension, heart disease and diabetes have been relatively well-developed. Patients with the chronic diseases can manage their conditions in a scientifically manner and control the progression of primary disease to prevent the secondary occurrence of AD resulting from the primary disease.

It should be noted that, due to constraints such as the inaccessibility of populations, endpoint indicators, and overly long observation periods, the evidence for the prevention of AD is mostly low level and has yet to be verified by randomized controlled trials (RCTs). Consequently, medical practitioners need to be cautious when using this data. There is an urgent need for large-scale population studies and RCTs to determine the preventive measures that may reduce the onset of AD in high-risk populations.

### 5.2 The preventive and regulatory roles of trace elements on AD

**Figure 3:**
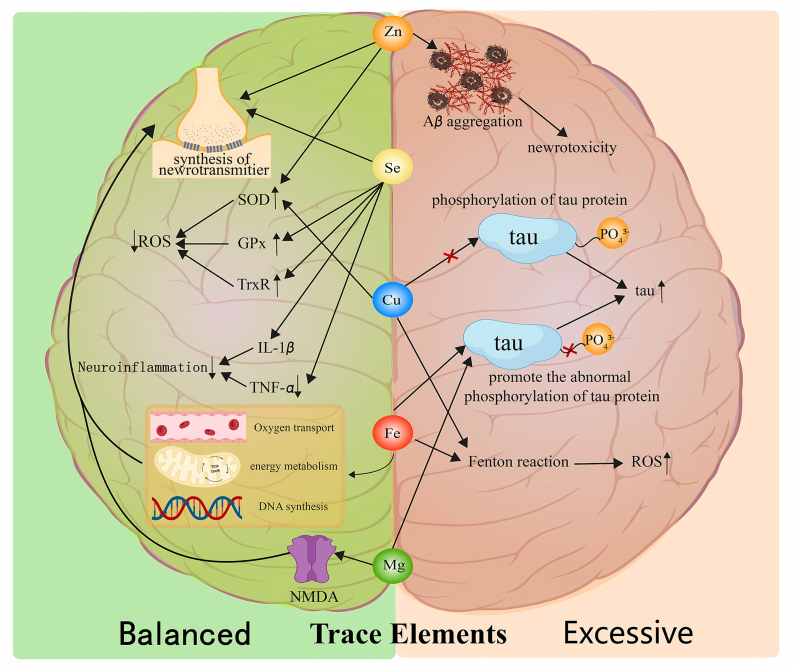
Dual roles of trace elements in Alzheimer’s disease.

Trace elements such as zinc, selenium, magnesium, copper and iron are not merely essential for maintaining the normal functionality of the nervous system but also play a crucial role in the pathophysiological mechanism of AD by participating in key biological processes such as antioxidant defense, anti-neuroinflammatory responses, synaptic plasticity, energy metabolism, and the regulation of gene expression. Dysregulated homeostasis of trace elements is implicated in AD pathology, where it may promote Abeta aggregation, exacerbate oxidative stress, and disrupt tau protein metabolism (Fig. 3). Although direct human data remain relatively limited, existing evidence points to distinct abnormalities in the cerebral distribution and metabolism of these elements in AD patients, highlighting their therapeutic potential^115^. Therefore, strategies aimed at maintaining trace element equilibrium, through dietary sources or supplementation, offer a promising and practical avenue for AD prevention.

#### 5.2.1 Zinc (Zn)

Zinc is an essential cofactor for many enzymes and a key modulator of neurotransmission in the brain. Its involvement in Alzheimer’s disease is complex and dualistic, encompassing both neuroprotective and pathology-promoting effects^116^^,^^117^. As a vital nutrient for the nervous system, zinc supports synaptic plasticity and neurotransmission, with notable impacts in hippocampal function^118^. Additionally, its role as a component of the antioxidant enzyme superoxide dismutase (SOD) positions zinc as a potential mitigator of the oxidative stress prevalent in AD^119^. Clinical observations consistently report disrupted zinc homeostasis in the AD brain, a disturbance linked to neuronal injury and cognitive impairment^120^. Preclinical findings further suggest that zinc supplementation can raise levels of brain-derived neurotrophic factor (BDNF) and improve cognitive performance^121^. Paradoxically, excess zinc or its dysregulation may worsen AD pathology; for example, zinc binding can induce conformational changes in Abeta, accelerating its aggregation and neurotoxicity. This dichotomy underscores zinc’s multifaceted nature and its potential as a therapeutic target, necessitating further research into its precise molecular mechanisms and homeostatic regulation^122^. For neurological health, maintaining adequate but not excessive zinc levels is crucial. A balanced diet featuring lean meats, seafood (*e*.*g*., oysters), legumes, nuts, and whole grains is the recommended source. In cases where supplementation is considered for at-risk individuals (*e*.*g*., the elderly), medical guidance is essential to prevent imbalances with other minerals like copper and iron.

#### 5.2.2 Selenium (Se)

Selenium has emerged as a trace element of considerable interest in Alzheimer’s research due to its potent antioxidant and anti-inflammatory functions, which are central to neuroprotection^123^. Its biological activity is primarily mediated through selenoproteins, such as glutathione peroxidase (GPx) and thioredoxin reductase (TrxR), where it is essential for scavenging reactive oxygen species (ROS) and preserving redox homeostasis^124^. Notably, studies report reduced selenium levels in the brains of AD patients, a deficit linked to increased oxidative stress and neuronal injury, partly through diminished GPx activity. Beyond its antioxidant role, selenium helps modulate neuroinflammation by suppressing pro-inflammatory cytokines like IL-1*β* and TNF-*α*^125^. These mechanisms suggest that restoring adequate selenium status could bolster antioxidant defenses and counter Abeta toxicity. Preclinical models support this notion, showing that selenium supplementation alleviates Abeta-driven neuroinflammation and cognitive deficits^126^. Preliminary clinical evidence also points to cognitive benefits from selenium in individuals with mild cognitive impairment (MCI)^127^. Interestingly, its role in mitigating oxidative stress may extend to related conditions, as seen in a double-blind trial where selenium improved outcomes in patients with migraine^128^. It is critical to note, however, that selenium has a narrow therapeutic window, and excessive intake carries risks of toxicity.

To support brain health, a diet inclusive of selenium-rich foods, such as Brazil nuts, fish, whole grains, and eggs, is recommended for the general population. For older adults or others with a suspected deficiency, supplementation should only be undertaken with clinical oversight to avoid adverse effects (*e*.*g*., hair loss, gastrointestinal issues, or neurotoxicity) from overconsumption. An integrated approach, pairing sensible nutrition with physical activity, sufficient sleep, and cognitive engagement, will likely yield the greatest benefit for long-term neurological health.

#### 5.2.3 Copper (Cu)

Copper exhibits a dual and complex relationship with AD: it is indispensable for normal neurophysiology, yet under conditions of dyshomeostasis, it can actively contribute to disease pathology^129^. As an essential enzymatic cofactor, for proteins such as cytochrome *c* oxidase and superoxide dismutase (SOD), copper is fundamental to cellular energy metabolism and antioxidant defense^130^. However, an imbalance in copper homeostasis is closely related to the pathological process of AD. Previous studies have shown that copper levels in the brains of patients with AD are abnormally elevated and that this may be related to the aggregation of beta-amyloid (Abeta) and enhanced neurotoxicity^131^. After binding with Abeta, copper promotes the formation of Abeta oligomers and generates ROS through catalytic oxidation reactions, exacerbating neuronal oxidative damage^132^. Furthermore, copper may promote the formation of neurofibrillary tangles by interfering with the phosphorylation of tau protein^133^. Despite this, an appropriate intake of copper is crucial for maintaining neurological function, and copper deficiency may lead to anemia, neurological dysfunction and weakened immune function.

A balanced diet is key to maintaining copper levels conducive to brain health, with shellfish, nuts, seeds, whole grains, and legumes serving as excellent dietary sources. Individuals with potential copper metabolism issues, including the elderly, should avoid excessive intake to prevent exacerbating oxidative stress and neurodegenerative pathology. Importantly, copper consumption must be considered in the context of overall mineral balance, due to its competitive absorption with elements like zinc and iron.

#### 5.2.4 Iron (Fe)

Iron metabolism represents a pivotal and complex factor in Alzheimer’s disease pathophysiology. While iron is indispensable as an enzymatic cofactor for fundamental processes like oxygen transport, energy metabolism, and DNA synthesis^134^, its dyshomeostasis is strongly implicated in AD pathogenesis^135^. Postmortem studies consistently show elevated iron levels in AD brains, where it is thought to potentiate neuronal oxidative damage and promote Abeta deposition^136^. Through the Fenton reaction, iron catalytically generates highly reactive hydroxyl radicals, driving significant oxidative injury to cellular lipids, proteins, and DNA^137^. Evidence also suggests that iron can facilitate the abnormal phosphorylation and aggregation of tau protein, accelerating the development of neurofibrillary tangles. It is important to recognize this dual nature: although iron excess can be pathological, sufficient dietary iron remains essential for preventing anemia and supporting baseline cognitive function.

To maintain optimal iron status, a balanced diet incorporating red meat, legumes, leafy green vegetables, and fortified grains is advisable. Individuals at risk of iron metabolism dysregulation, including older adults, should avoid excessive intake to minimize potential aggravation of oxidative stress and neurodegenerative processes. Consuming iron alongside vitamin C-rich foods can improve its absorption.

#### 5.2.5 Magnesium (Mg)

Magnesium has gained recognition as a key neuroprotective agent, with growing evidence linking its status to cognitive health and Alzheimer’s disease progression^138^. This essential cation, among the most abundant in the body, supports a wide array of biological functions, from energy metabolism and DNA repair to neurotransmission^139^. Its influence extends to synaptic plasticity and learning, mediated in part through modulation of NMDA receptor activity^140^. Notably, AD is associated with significantly reduced brain magnesium levels, a deficit that may underlie aspects of cognitive decline and neurodegeneration^141^. Magnesium deficiency can also trigger mitochondrial impairment and oxidative stress, creating conditions that favor both Abeta production and tau hyperphosphorylation^142^. Conversely, replenishing magnesium appears to confer protective effects, bolstering synaptic resilience and dampening neuroinflammation. Preclinical studies support this, demonstrating that magnesium supplementation improves cognition and reduces Abeta deposition in AD mouse models^143^. Early clinical data are promising, suggesting that magnesium may enhance memory and executive function in individuals with mild cognitive impairment^144^. As with many interventions, balance is critical; excessive magnesium intake can cause gastrointestinal upset or electrolyte disturbances, underscoring the need for careful dosing.

For the general public, adequate magnesium supplementation may have positive implications for maintaining brain health. A balanced diet is recommended for acquiring magnesium. Foods rich in magnesium include green leafy vegetables (such as spinach), nuts, legumes and whole grains. For the elderly or people at risk of magnesium deficiency, magnesium supplementation can be used under the guidance of a doctor; however, excessive intake should be avoided to avoid diarrhea or electrolyte imbalance. In addition, magnesium supplementation should be combined with a healthy lifestyle, including regular exercise, adequate sleep and cognitive training for overall brain health.

In addition to the preventive and therapeutic effects of individual trace elements on AD, recent research has indicated that the combinations of two or more trace elements might exert a synergistic effect on the prevention and treatment of AD^145^. For example, the combination of zinc and selenium has been shown to exert a stronger antioxidant and anti-inflammatory action in animal experiments, significantly reducing the deposition of Abeta and improving cognitive function^146^. In addition, the combined supplementation of magnesium and zinc has been demonstrated to synergistically enhance synaptic plasticity and reduce neuronal damage in cell models by regulating the functionality of NMDA receptors^147^. Clinical studies also suggest that the balanced supplementation of multiple trace elements might be more efficacious in enhancing the cognitive function of patients with MCI than single elements. Supporting this concept, a clinical intervention in an elderly cohort demonstrated that combined supplementation with zinc, selenium, and magnesium significantly improved cognitive scores and lowered levels of key AD-related biomarkers^148^. This finding suggests that trace element combinations can simultaneously engage multiple pathological pathways in AD, leading to greater overall benefit than single-element approaches. To translate this potential into practical strategies, future work must determine the optimal ratios of these elements and elucidate the precise molecular mechanisms underlying their synergy, thereby enabling the design of more effective, multi-targeted nutritional interventions for AD.

In summary, trace elements are integral to both the physiological functioning and the pathophysiological cascade of AD, with their strict homeostatic balance being crucial for neuronal health. Adequate intake supports key neuroprotective processes, including antioxidant defense, synaptic plasticity, and energy metabolism, and may therefore favorably influence AD prevention and management. Critically, this balance is delicate; dysregulation through either deficiency or excess can disrupt homeostasis and exacerbate central AD pathologies like oxidative stress, Abeta aggregation, and tau hyperphosphorylation. A varied and balanced diet is the foundational approach to securing sufficient trace elements for brain health. When supplementation is warranted, it requires professional medical supervision to avoid toxicity or interactions with other minerals. Advancing this field necessitates deeper mechanistic insights into how trace elements modulate AD pathways, with the ultimate goal of devising homeostasis-informed, precise nutritional interventions.

### 5.3 The preventive and regulatory effects of vitamins on AD

**Figure 4:**
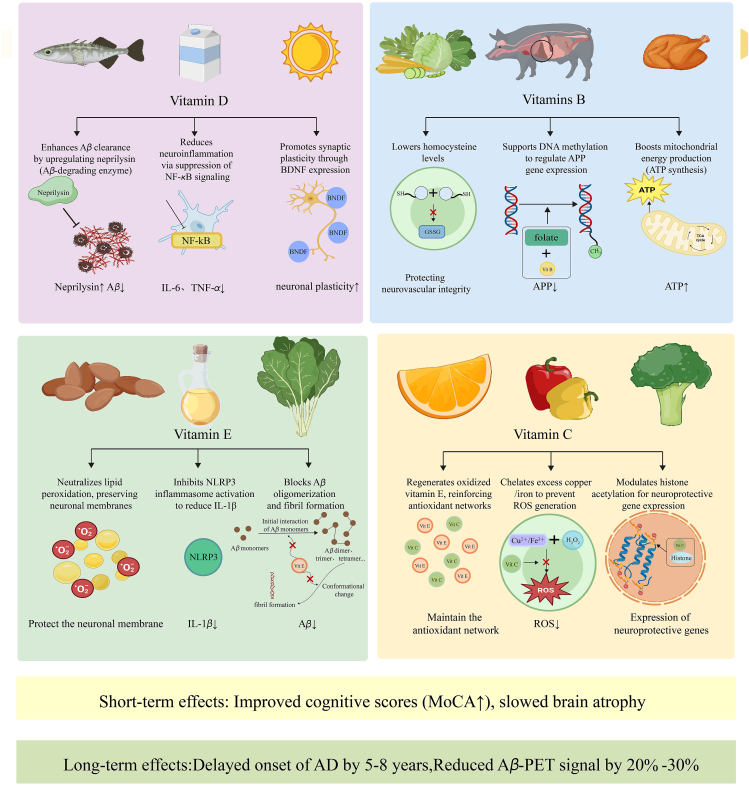
Vitamins in Alzheimer’s disease prevention.

The contribution of vitamins to neurological health is well-established, and their potential to influence Alzheimer’s disease pathways is an area of growing research interest. A body of evidence now points to specific roles for vitamins in supporting antioxidant defenses, modulating neuroinflammatory responses, and maintaining synaptic plasticity, key processes that offer viable targets for AD prevention strategies (Fig. 4).

#### 5.3.1 Vitamin D

Vitamin D, a fat-soluble secosteroid, exerts pleiotropic effects critical for maintaining calcium homeostasis and modulating immune responses. In addition to their classical functions, vitamin D receptors are widely expressed in the hippocampus and cortex, implicating their role in cognitive processes^149^. Epidemiological studies indicate that vitamin D deficiency is associated with a 1.5- to 2-fold increased risk of AD^150^. Mechanistically, vitamin D attenuates Abeta toxicity by upregulating neprilysin, a key Abeta-degrading enzyme, and reduces tau hyperphosphorylation *via* the inhibition of glycogen synthase kinase-3beta (GSK-3beta)^151^. A meta-analysis of 12 prospective cohorts demonstrated that adequate serum levels of 25-hydroxyvitamin D (>30 ng/mL) correlated with a 33% lower incidence of dementia^152^. Animal studies further revealed that vitamin D supplementation in AD models ameliorated synaptic loss and improved spatial memory by enhancing the expression levels of BDNF^153^. For the general population, a daily intake of 800–2000 IU of vitamin D through sunlight exposure, fatty fish or fortified foods is recommended. However, excessive supplementation (>4000 IU/day) may lead to hypercalcemia, necessitating medical supervision^154^^,^^155^.

#### 5.3.2 Vitamin B complex

The B-vitamin group, particularly folate (B9), vitamin B12, and pyridoxine (B6), is critical for homocysteine metabolism. Elevated plasma levels of homocysteine (>14 μmol/L) is a recognized risk factor for AD and can promote oxidative stress and vascular endothelial dysfunction^156^. Vitamins B12 and folate are essential cofactors in the conversion of homocysteine to methionine, a remethylation process that limits its neurotoxic buildup. Supporting this importance, a randomized controlled trial of 1202 older adults demonstrated that combined B6/B9/B12 supplementation reduced the rate of hippocampal atrophy by 30% in participants with elevated baseline homocysteine (>11 μmol/L)^157^. Separately, vitamin B12 deficiency may worsen AD pathology; studies in APP/PS1 mouse models indicate that impaired methylmalonyl-CoA mutase activity, a B12-dependent enzyme, can exacerbate mitochondrial dysfunction and Abeta deposition^158^. To support healthy homocysteine levels, dietary intake from sources like leafy greens, eggs, and lean meats is recommended. High-dose supplemental B12 (>1 mg/day) is generally not advised without medical supervision, as it may interfere with certain medications^159^.

#### 5.3.3 Vitamin E

As a major lipid-soluble antioxidant, vitamin E (primarily as alpha-tocopherol) helps protect neuronal membranes from oxidative damage. This protection appears compromised in AD, where postmortem analyses show reduced brain levels of vitamin E alongside elevated markers of lipid peroxidation (*e*.*g*., 4-hydroxynonenal)^160^. Clinically, high-dose alpha-tocopherol supplementation (2000 IU/day) showed benefit in the 2023 TEAM-AD trial, slowing functional decline by 19% over two years in mild-to-moderate AD; a proposed mechanism involves the inhibition of the NLRP3 inflammasome^161^. Beyond *α*-tocopherol, preclinical research indicates that the vitamin E isoform *γ*-tocotrienol may promote Abeta clearance *via* enhanced microglial phagocytosis^162^. Dietary intake from nuts, seeds, and vegetable oils is recommended to maintain adequate levels. However, due to an increased hemorrhage risk associated with prolonged high-dose intake (>1000 IU/day), caution is advised, particularly for individuals on anticoagulant therapy^163^.

#### 5.3.4 Vitamin C

A core function of vitamin C (ascorbic acid) in neuroprotection is its synergistic action with vitamin E, which amplifies the scavenging of ROS and facilitates the recycling of reduced glutathione. However, the role of vitamin C in AD does not just involve antioxidant activity; vitamin C inhibits Abeta oligomerization by chelating metal ions (*e*.*g*., copper and iron) and enhances synaptic plasticity *via* the epigenetic regulation of NMDA receptors^164^. A longitudinal study of 13,375 participants found that a high dietary intake of vitamin C (≥110 mg/day) was associated with a 24% lower incidence of AD over a decade^165^. Animal studies, using APPswe/PS1dE9 mice, revealed that vitamin C supplementation restored hippocampal neurogenesis and reduced neuroinflammation by suppressing NF-*κ*B signaling^166^. Citrus fruits, bell peppers and broccoli are optimal sources of vitamin C, with a recommended daily intake of 75–90 mg for adults. However, an excessive intake (>2000 mg/day) may cause gastrointestinal distress or kidney stones^167^.

#### 5.3.5 Synergistic effects of vitamin combinations

Targeting Alzheimer’s disease through combined vitamin supplementation is gaining traction, as this approach can simultaneously address several pathological pathways. Clinical evidence supports this strategy; one randomized trial found that co-administering vitamins D and E boosted cognitive scores by 18% in MCI patients compared to single-vitamin regimens, likely through complementary anti-inflammatory and antioxidant actions^168^. Mechanistically, the interaction between B vitamins and vitamin C can reduce vascular injury from homocysteine while also strengthening the body’s intrinsic antioxidant capacity^169^. This collective evidence points toward personalized multivitamin strategies, where formulations are adjusted based on an individual’s nutritional biomarkers and genetic risk, such as APOE *ε*4 carrier status^170^.

In summary, vitamins influence AD through diverse mechanisms, including antioxidation, metabolic control, and neuroimmune regulation. Although existing studies underscore their preventive value, defining optimal doses and confirming long-term safety will depend on more extensive, well-controlled clinical trials. For populations at elevated AD risk, a pragmatic preventive approach may involve combining evidence-based vitamin supplementation with broader lifestyle interventions.

### 5.4 The preventive and regulatory effects of physical exercise on AD

Among non-pharmacological interventions for AD, regular physical activity is perhaps the most versatile and accessible. It delivers direct neuroprotective effects while also reducing the burden of common age-related conditions like cardiovascular disease, diabetes, and obesity, each a known modifier of AD risk. A growing body of research delineates how exercise strengthens cognitive reserve and postpones clinical AD onset *via* multiple, interrelated biological pathways^171^.

#### 5.4.1 Neurobiological mechanisms

The neurobiological benefits of exercise are multi-pronged. It upregulates brain-derived neurotrophic factor (BDNF), a protein essential for neuronal survival, synaptic plasticity, and the birth of new neurons^172^. Aerobic activities, such as brisk walking or cycling, are especially effective at increasing cerebral blood flow and have been shown to stimulate growth in hippocampal volume, directly opposing the region-specific atrophy that characterizes early AD^173^. At a systemic level, exercise dampens chronic inflammation and oxidative stress, key drivers of AD pathology, by reducing circulating levels of pro-inflammatory cytokines like IL-6 and TNF-*α*^174^. These mechanistic insights are corroborated by animal studies, where regular physical activity reduces cerebral Abeta plaque load and improves memory in AD transgenic mice, effects linked to improved mitochondrial efficiency and cellular clearance mechanisms^175^.

#### 5.4.2 Clinical and epidemiological evidence

Epidemiological evidence robustly supports the protective role of exercise. A large meta-analysis encompassing 45 prospective studies and over 1.2 million participants concluded that individuals who engaged in at least 150 min of moderate-to-vigorous activity weekly had a 35% lower risk of developing AD than their sedentary peers^176^. Consistent with this, the 2024 World Health Organization guidelines affirm that even low-intensity activities, including walking and tai chi, can meaningfully slow cognitive decline in older adults, with benefits accruing when such habits are established by midlife^177^. Neuroimaging corroborates these population-level findings, showing that sustained physical activity helps preserve gray matter density in brain regions highly vulnerable to AD, including the prefrontal and entorhinal cortices^178^.

#### 5.4.3 Synergistic benefits for the prevention of multimorbidity

The value of exercise extends beyond AD-specific pathology to modify shared risk factors for age-related decline. For example, by improving insulin sensitivity, regular activity lowers the incidence of type 2 diabetes, a recognized contributor to AD risk^179^. Resistance training, in turn, counteracts age-related sarcopenia and osteopenia, thereby reducing frailty and the risk of injurious falls. This capacity to concurrently address multiple geriatric syndromes positions physical exercise as a uniquely cost–effective and scalable public health intervention for aging societies.

#### 5.4.4 Practical recommendations

Practical guidelines recommend a mixed regimen of aerobic exercise (*e*.*g*., brisk walking), strength training, and balance activities for comprehensive cognitive protection. Importantly, benefits are still attainable with fragmented activity; for older adults, short bouts such as two 10-min walks per day can produce measurable gains^180^. Adherence, which many people find challenging, can be strengthened through community-based programs like group classes or walking clubs. These activities cultivate social connection, an important protective factor for cognitive health, and establish supportive environments where individuals feel motivated and included.

In conclusion, physical exercise represents a foundational, universally applicable non-pharmacological strategy for AD prevention. Its power lies in this dual ability: to directly counteract AD pathophysiology and to ameliorate the systemic aging processes that fuel it. Therefore, integrating the promotion of lifelong physical activity into public health policy and clinical practice must be a priority for mitigating the rising global burden of AD.

### 5.5 Staging of AD and its impact on prevention

The revised 2024 NIA-AA diagnostic criteria for AD establish a dual staging framework, categorizing the illness along biological (stages A–D) and clinical (stages 0–6) axes^43^. Biological stages track the advancement of core pathologies: Abeta deposition, tau aggregation, and neurodegeneration. In parallel, clinical stages quantify the severity of cognitive and functional impairment, ranging from asymptomatic to profound dementia. This refined staging system enables a more nuanced understanding of AD progression and facilitates the design of stage-specific preventive interventions.

During the earliest phase (Stage 0–1), individuals are asymptomatic but may harbor elevated risk from genetic predisposition or positive biomarker status, such as brain amyloid accumulation. Preventive efforts here are primarily population-based and focus on mitigating modifiable risks: controlling hypertension and LDL cholesterol, preventing diabetes and obesity, promoting smoking cessation, and addressing sensory deficits like hearing and vision loss^181^. For those identified as high-risk through genetic screening or biomarker testing, this stage also offers a critical window for initiating targeted strategies to lower long-term AD probability.

Stage 2 describes subjective cognitive decline, where individuals self-report worsening memory or thinking skills despite normal performance on standardized cognitive tests. Interventions at this juncture should emphasize comprehensive lifestyle modification. Proven strategies include regular physical activity, structured cognitive training, and adherence to neuroprotective dietary patterns like the Mediterranean or MIND diets. Supplementation with specific nutrients or vitamins and efforts to minimize psychosocial stressors may provide additional support to bolster cognitive resilience and slow progression.

Stage 3, or mild cognitive impairment (MCI) due to AD, involves objective cognitive deficits that fall short of dementia. Management at this stage integrates sustained lifestyle interventions with a clinical evaluation for potential disease-modifying therapies (DMTs). Pharmacological options may include cholinesterase inhibitors or anti-amyloid biologics to slow decline. Crucially, aggressive management of comorbid vascular risk factors, including hypertension and diabetes, continues to be a cornerstone of care for stabilizing cognitive function^182^^,^^183^.

In Stages 4 through 6, encompassing mild to severe dementia, cognitive and functional impairments are pronounced, leading to increasing dependence. The goal of prevention shifts to a secondary focus: preserving safety, maximizing remaining functional capacities, and optimizing quality of life. Care is predominantly symptomatic, involving pharmacological management of cognitive and neuropsychiatric symptoms (*e*.*g*., depression, anxiety, agitation), alongside physical and occupational therapy to maintain daily skills. Strong caregiver support systems are essential^184^.

This staging paradigm underscores the progressive nature of AD and the consequent necessity for dynamically tailored preventive approaches. Utilizing the revised NIA-AA criteria to identify disease stage allows for the application of the most appropriate interventions, from primary prevention in asymptomatic at-risk individuals to supportive and symptomatic management in those with advanced dementia.

### 5.6 Sex-based differences in AD pathogenesis and preventive strategies

Biological sex critically influences both susceptibility to AD and the potential efficacy of its prevention^185^^,^^186^. Women, especially after menopause, show a higher incidence of AD. This disparity is linked to the loss of neuroprotective hormones like estrogen, which adversely affects synaptic integrity, mitochondrial efficiency, and Abeta clearance. Furthermore, the APOE *ε*4 allele, a major genetic risk factor, imposes a greater detrimental effect in women, accelerating hippocampal atrophy and cognitive decline in female carriers compared to males^187^^,^^188^. In men, however, AD risk appears more strongly tied to vascular and metabolic health; conditions like hypertension and diabetes confer a proportionally higher relative risk for AD in male populations, pointing to distinct pathogenic avenues. Consequently, optimal preventive approaches should be sex-informed. For women, hormone replacement therapy initiated around the perimenopausal transition may lower AD risk, though its net benefit depends heavily on timing, formulation, and individual health profile. For men, primary prevention may more effectively focus on the rigorous control of cardiometabolic risk factors. While foundational lifestyle interventions, such as physical activity and a nutritious diet, remain universally recommended, their protective impact may vary in degree based on an sex-specific hormonal and genetic context^189^. Therefore, developing effective prevention frameworks necessitates a nuanced integration of sex, genetic predisposition, hormonal status, and comorbid condition.

## 6 Discussion

Alzheimer’s disease is a progressive neurodegenerative condition that places severe physical, emotional, and financial strain not only on patients but also on their family networks. Simultaneously, it exerts significant pressure on healthcare infrastructures and broader socioeconomic systems globally. As demographic aging accelerates worldwide, AD prevalence climbs yearly, deepening its societal footprint. While therapeutic research has achieved notable advances in recent years^190^^,^^191^, existing clinical interventions still cannot effectively stop or reverse the disease trajectory, and treatment results often remain disappointing^192^^,^^193^. Therefore, shifting focus toward preventing AD onset and slowing its advancement has become an indispensable approach for reducing the overall burden it creates.

Research dedicated to AD prevention, however, confronts several formidable obstacles. A primary issue is the protracted natural history of the disease; pathological changes often commence decades prior to symptom onset. This reality demands prevention studies to adopt lengthy follow-up periods and enroll large population cohorts, inevitably leading to extended project durations and substantial financial investment. Furthermore, assessing the success of preventive measures is intrinsically difficult. Conventional short-term quantitative metrics may fail to capture meaningful signals, for example, early, subtle cognitive shifts or minor biomarker variations might not reach statistical significance within brief observation windows. These methodological complexities hinder robust data collection and interpretation, and they also slow the translation of research into high-impact journal publications. As a direct result, scientists working in prevention frequently encounter greater hurdles in obtaining competitive research grants.

In light of both the clear public health imperative and the distinctive methodological demands of prevention science, a strategic reassessment of funding priorities by governmental and regulatory agencies is warranted. Enhanced and sustained support for AD prevention research is critically needed. Concrete steps should include creating dedicated funding streams specifically designed to facilitate the long-term, large-scale cohort studies that this field requires, and to accelerate the refinement of more sensitive tools for early detection, including novel biomarkers and cognitive assays. Equally important is evolving the criteria used to evaluate such research impact. Moving beyond a narrow reliance on publication metrics alone, funding bodies should recognize and reward the practical, real-world benefits and the long-term scientific value inherent in prevention studies, even if their academic outputs unfold over many years. It is only through such committed policy backing and strategic resource allocation that transformative breakthroughs in AD prevention can be realized, finally mitigating the heavy dual burden this disease imposes on both individuals and society.

**Figure 5:**
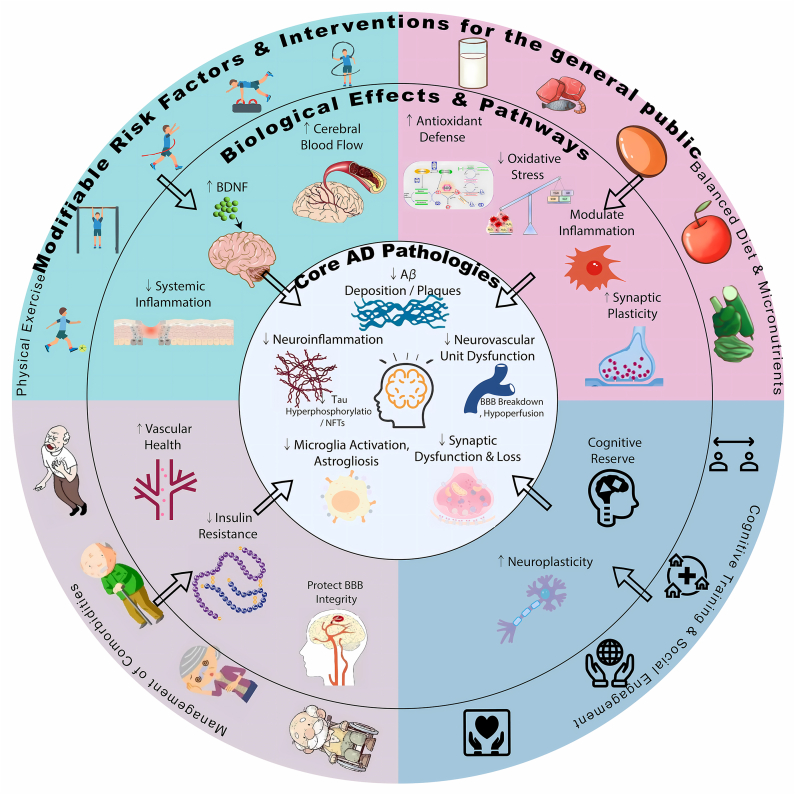
Mechanistic interactions between modifiable risk factors, biological pathways, and core AD pathologies.

From a public health perspective, modifying daily lifestyle habits stands out as the most accessible and sustainable strategy for AD risk reduction (Fig. 5). Compelling evidence suggests that interventions addressing several modifiable risk factors in concert can significantly lower the probability of developing the disease. One key avenue is ensuring a balanced intake of specific trace elements. Zinc, selenium, and magnesium, for instance, play crucial protective roles in brain health. Their benefits are mediated through diverse mechanisms, such as modulating oxidative stress, hindering the aggregation of Abeta proteins, and supporting synaptic function. To achieve optimal levels of these nutrients, individuals are advised to consume a diverse diet. Regularly incorporating foods like nuts, fish, dark leafy greens, and whole grains can help meet nutritional needs naturally, thereby circumventing the potential imbalances associated with indiscriminate supplement use. Secondly, consistently following dietary patterns recognized for their brain-health benefits, like the Mediterranean or MIND diets, supplies a wealth of antioxidants (including vitamins C and E) and anti-inflammatory agents. These dietary components help counteract neuroinflammation and the associated cognitive deterioration. Complementing nutrition, regular engagement in aerobic physical activity, meeting or exceeding 150 min weekly, boosts blood flow to the brain, supports the preservation of hippocampal volume, and strengthens synaptic plasticity, partly by elevating levels of brain-derived neurotrophic factor (BDNF). The strategic management of trace element intake offers a potent, diet-based method for the public to influence AD risk. Zinc exemplifies the nuanced approach required. Its involvement in AD pathology is complex and dual-sided^194^^,^^195^. Insufficient zinc can detrimentally affect synaptic plasticity and dampen BDNF production, raising vulnerability to cognitive deficits^196^. Paradoxically, an oversupply of zinc might encourage Abeta proteins to bind together and form toxic oligomers more rapidly, thus amplifying neuronal damage^197^. This dichotomy clearly indicates that public health advice and clinical guidance must center on achieving zinc homeostasis, a balanced state, rather than advocating for generalized, unregulated supplementation. For instance, obtaining zinc through a well-balanced diet, including sources such as seafood, legumes, and whole grains, is generally advantageous. However, high-dose zinc supplements should be limited to individuals with confirmed deficiencies. Moreover, zinc supplementation should be accompanied by the monitoring of copper and iron levels to prevent potential imbalances due to competitive absorption^198^. Therefore, navigating zinc’s ‘dual role’ calls for a personalized prevention tactic. This strategy should aim for sufficient, but not surplus, intake and must be calibrated according to a person’s specific nutritional profile and any co-existing health conditions.

For individuals managing chronic conditions like hypertension or diabetes, effectively controlling blood pressure, glucose, and lipid levels can substantially reduce the risk of vascular-related cognitive impairment. A key point is that these strategies demand long-term commitment and integration into daily routines; they should not be viewed as temporary fixes. A growing consensus in the literature indicates that no single preventive approach works universally for all populations. Each intervention has its own profile of benefits and drawbacks, and its success depends heavily on the stage of disease, an individual’s baseline risk factors, and their adherence to the regimen. Consider intensive blood pressure control: while it offers robust benefits for cardiovascular and cognitive health, its application in frail elderly patients requires caution due to risks of hypotension and renal impairment. Hearing interventions demonstrate the strongest protective effect for individuals already at high risk for dementia, but the benefit is less pronounced in general population cohorts. Multidomain lifestyle programs, exemplified by the FINGER and U.S. POINTER trials, achieve broad cognitive gains, yet they require significant resources and a high degree of participant engagement. Modifying diet, particularly adopting Mediterranean-style patterns, can modulate metabolic and genetic risks, though evidence from randomized trials on cognitive outcomes remains mixed. Recent public health guidance underscores the importance of lipid management in midlife and vision care in later life, while generally discouraging the routine use of nutritional supplements for dementia prevention. A comparative analysis of these preventive strategies is presented in Table 4, which details their target populations, primary cognitive outcomes, advantages, and limitations. This comparison underscores a critical need for stratified and individualized prevention plans. Tailoring interventions based on factors such as age, sex, genetic risk profile, and comorbidity status can optimize efficacy and minimize potential adverse effects^199^^,^^200^. It is clear that while individual benefits may vary, the cumulative and synergistic impact of multidimensional lifestyle changes can markedly slow the progression of AD pathology. Consequently, a priority for public health policy should be to foster awareness and adoption of these preventive measures through education and community-based initiatives, thereby alleviating the societal burden imposed by AD.

**Table 4:** Preventive strategies: applicability, advantages, disadvantages, and representative evidence.

Strategy	Stage/population	Outcome	Advantage	Limitation
Intensive BP control^201^	Midlife–late life; hypertensive; stage 0–3	SPRINT-MIND: ↓ MCI (HR 0.81); ↓ MCI + probable dementia (HR 0.85)	CVD benefit; widely implementable	Hypotension, AKI risk
Hearing intervention^202^	Older adults with hearing loss; higher-risk subgroup	Achieve: ∼50% slower decline in high-risk subgroup; overall neutral	Improves communication/QOL	Adherence; benefit mainly in high-risk subgroup
Multidomain lifestyle^203^	At-risk adults (CAIDE≥6; APOE *ε*4); stage 0–2	FINGER: +25% cognition; U.S. POINTER: Structured > control	Broad cardiometabolic benefits	Resource intensive
Diet (MIND/Mediterranean)^204^^,^^205^	General older adults; APOE *ε*4 carriers; stage 0–2	NEJM 2023 MIND-diet RCT: Neutral; Nat Med 2025: MeDi offsets genetic risk	Low risk; aligns with CVD prevention	Heterogeneity; RCT superiority not shown
LDL management^206^	Midlife dyslipidemia; stage 0–2	Lancet Commission 2024: PAF ≈7%	Established CVD benefit	Dementia-specific evidence limited
Vision care^206^^,^^207^	Late-life vision impairment; stage 0–2	Lancet Commission 2024: PAF ≈2%	Improves function/safety	Evidence mostly observational
Supplements	General older adults	WHO guideline: no benefit of routine vitamins/omega-3	Easy to implement	No cognitive benefit

BP, blood pressure; CVD, cardiovascular disease; HR, hazard ratio; MCI, mild cognitive impairment; PAF, population-attributable fraction; MeDi, Mediterranean diet; WHO, World Health Organization.

## 7 Conclusions

Currently, therapeutic strategies for Alzheimer’s disease focus on multiple pathological pathways. These include amyloid-beta aggregation, tau pathology, neuroinflammation, and synaptic dysfunction. Available treatments, ranging from monoclonal antibodies to cholinesterase inhibitors, can slow disease progression but do not reverse established neuronal damage. Although several ongoing randomized controlled trials are evaluating novel approaches such as tau-targeted therapies and anti-inflammatory agents, their clinical benefits remain uncertain. In contrast, lifestyle modifications offer a widely accessible and practical avenue for prevention. Consistent daily habits contribute significantly to risk reduction: a balanced diet that provides adequate zinc, selenium, and vitamin D; regular physical exercise; and careful management of vascular risk factors such as hypertension and diabetes. These measures are associated with a lower incidence of AD and delayed cognitive decline. Because they are cost–effective, low-risk, and easily incorporated into routine life, they represent an ideal strategy for primary prevention, especially in aging and high-risk populations. Moving forward, research should continue to validate the synergistic effects of multidimensional lifestyle interventions. Equally important are policy initiatives that better integrate preventive medicine with public health infrastructure. Translating scientific evidence into individual behavior change and supportive societal systems will be essential to mitigate the growing global burden of AD.

## Author contributions

Gao Zhonggao: Conceptualization, Data curation, Funding acquisition, Project administration, Resources, Supervision, Validation, Writing – review & editing. Lili Jin: Formal analysis, Writing – original draft, Funding. Wenyu Jin: Formal analysis, Methodology, Funding. Mingfeng Han: Data curation, Formal analysis, Investigation, Methodology, Visualization, Writing – original draft. Nuoya Wang: Formal analysis, Funding acquisition, Resource. Mengxiu Song: Formal analysis, Resource, Funding. Chenfei Liu: Funding acquisition. Yedan Wu: Data curation, Jishan Ying: Resource.

## Declaration of generative AI in scientific writing

During the preparation of this work the authors used ChatGPT (OpenAI) in order to improve readability and grammar. After using this tool/service, the authors reviewed and edited the content as needed and take full responsibility for the content of the publication.

## Conflicts of interest

The authors declare no conflict of interest.
